# Absent or Low Rate of Adult Neurogenesis in the Hippocampus of Bats (Chiroptera)

**DOI:** 10.1371/journal.pone.0000455

**Published:** 2007-05-23

**Authors:** Irmgard Amrein, Dina K.N. Dechmann, York Winter, Hans-Peter Lipp

**Affiliations:** 1 University of Zurich, Institute of Anatomy, Division of Neuroanatomy and Behavior, Zurich, Switzerland; 2 Department of Biology, University of Munich, Munich, Germany; James Cook University, Australia

## Abstract

Bats are the only flying mammals and have well developed navigation abilities for 3D-space. Even bats with comparatively small home ranges cover much larger territories than rodents, and long-distance migration by some species is unique among small mammals. Adult proliferation of neurons, i.e., adult neurogenesis, in the dentate gyrus of rodents is thought to play an important role in spatial memory and learning, as indicated by lesion studies and recordings of neurons active during spatial behavior. Assuming a role of adult neurogenesis in hippocampal function, one might expect high levels of adult neurogenesis in bats, particularly among fruit- and nectar-eating bats in need of excellent spatial working memory. The dentate gyrus of 12 tropical bat species was examined immunohistochemically, using multiple antibodies against proteins specific for proliferating cells (Ki-67, MCM2), and migrating and differentiating neurons (Doublecortin, NeuroD). Our data show a complete lack of hippocampal neurogenesis in nine of the species *(Glossophaga soricina, Carollia perspicillata, Phyllostomus discolor, Nycteris macrotis, Nycteris thebaica, Hipposideros cyclop*s, *Neoromicia rendalli, Pipistrellus guineensis*, and *Scotophilus leucogaster*), while it was present at low levels in three species (*Chaerephon pumila, Mops condylurus* and *Hipposideros caffer*). Although not all antigens were recognized in all species, proliferation activity in the subventricular zone and rostral migratory stream was found in all species, confirming the appropriateness of our methods for detecting neurogenesis. The small variation of adult hippocampal neurogenesis within our sample of bats showed no indication of a correlation with phylogenetic relationship, foraging strategy, type of hunting habitat or diet. Our data indicate that the widely accepted notion of adult neurogenesis supporting spatial abilities needs to be considered carefully. Given their astonishing longevity, certain bat species may be useful subjects to compare adult neurogenesis with other long-living species, such as monkeys and humans, showing low rates of adult hippocampal neurogenesis.

## Introduction

Bats (Chiroptera) are the only mammals capable of active flight and, together with marine mammals (Cetacea), navigate effortlessly in their three-dimensional environments. Particularly fruit- and nectar-feeding species benefit from a precise spatio-temporal memory to relocate profitable food sources at flowering or fruiting plants [1]. In rodents and bats, the hippocampus is thought to process spatio-temporal relationships as indicated by the presence of neurons active during spatial behavior (“place cells”) [2], [3], the human hippocampus is also required for establishing episodic memory. In agreement with this view, the hippocampus of fruit- and nectar-feeding bat species is enlarged relative to the size of the remaining hemispheres [4], [5].

**Figure 1:**
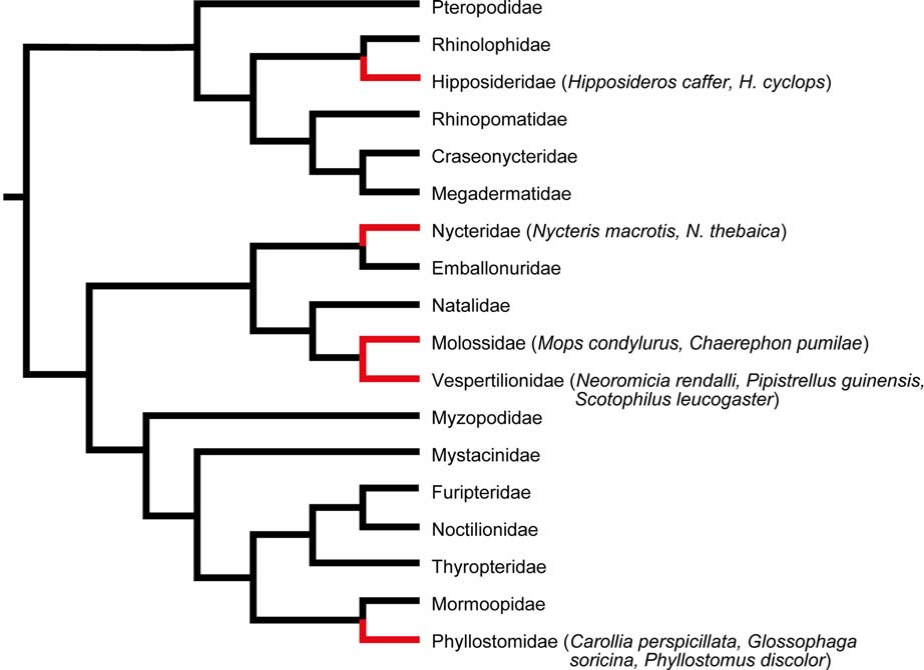
Phylogenic tree of all extant bat families. Names of species included in this study in brackets behind the corresponding family (adapted from Teeling et al. 2005 [43]).

Adult proliferation of neurons in mammals is thought to be restricted to two regions: a subventricular zone (SVZ) at the rostral end of the lateral ventricles, from where newly formed cells migrate towards the olfactory bulb, and a narrow zone below the granule cells of the dentate gyrus in the hippocampus that forms an integral part of the circuitry of this brain region. Proliferation of these progenitor cells is coined adult hippocampal neurogenesis (AHN), although this term also includes cells that later become glial cells. Studies in rodents have shown that such newly formed neurons integrate into the existing cells, form connections and show electrical activity [6]. Likewise, rodent studies have found that proliferation rate and survival of newly born cells is under physiological regulation, the most replicated findings being increased proliferation after voluntary physical exercise [7], and suppression of neurogenic activity by psychological stress [8]. It is also commonly recognized that AHN declines with age, and that cell proliferation may occur reactively after injuries or pathological processes affecting the brain [9]. Two lines of research have emerged. One aims to understand AHN and its relevance for restoring brain functions, particularly in humans, the other is searching for the functional role of AHN in the normal brain. The latter approach has largely focused on demonstrating relations between experimentally altered AHN and spatial learning abilities of rodents, variations in neurogenesis being taken as a marker for hippocampal functions. However, experimental rodent data about the functional relevance of hippocampal neurogenesis for learning and memory are contradictory, resulting just as often in a total lack of evidence as in positive findings [10]. A possible shortcoming of many previous studies is that small laboratory test arenas might not be sufficient to trigger plasticity mechanisms that evolved for coping with natural large-scale orientation requirements typical for wild living animals. This interpretation is offered by studies where neurogenesis was determined for wild living rodent species whose learning experiences had taken place through natural activities in their ecological environments [11], [12]. Here, rates of neurogenesis correlated very clearly with the ecological requirements for spatial orientation ability. Since the species with the largest territory (*Apodemus flavicollis*) also had the longest life span, the high levels of AHN in these mice might counterbalance the age-related decline, offering minimal functional levels of AHN even at advanced age. From these results based on rodents, we predicted that high rates of neurogenesis should also be expected in wild living bats with high ecological demands on their spatial orientation abilities, the latter being moreover required throughout their long life span. The present study was therefore conducted with the intent to broaden the empirical basis of the previous findings, and to examine to which degree the findings in rodents can be extrapolated to other species. We investigated twelve tropical species belonging to five different families (Fig. 1), comprising three nectar- and fruit eating Neotropical and nine Paleotropical insectivorous species with different foraging strategies. Proliferating cells were identified with antibodies against Ki-67, a chromosome-associated protein present in the active phase of the cell cycle [13], and MCM2, a licensing factor for cell division [14]. Young migrating or differentiating neurons were detected with antibodies against Doublecortin (DCX), a microtubule-associated protein present in migrating neuroblasts and during maturation of developing neurons [15], and NeuroD, a transcription factor regulating neuronal fate [16]. Contrary to our expectation, however, we did not find the predicted high rates of hippocampal neurogenesis.

## Results

### No proliferating cells in the hippocampus in nine of twelve bat species

**Figure 2:**
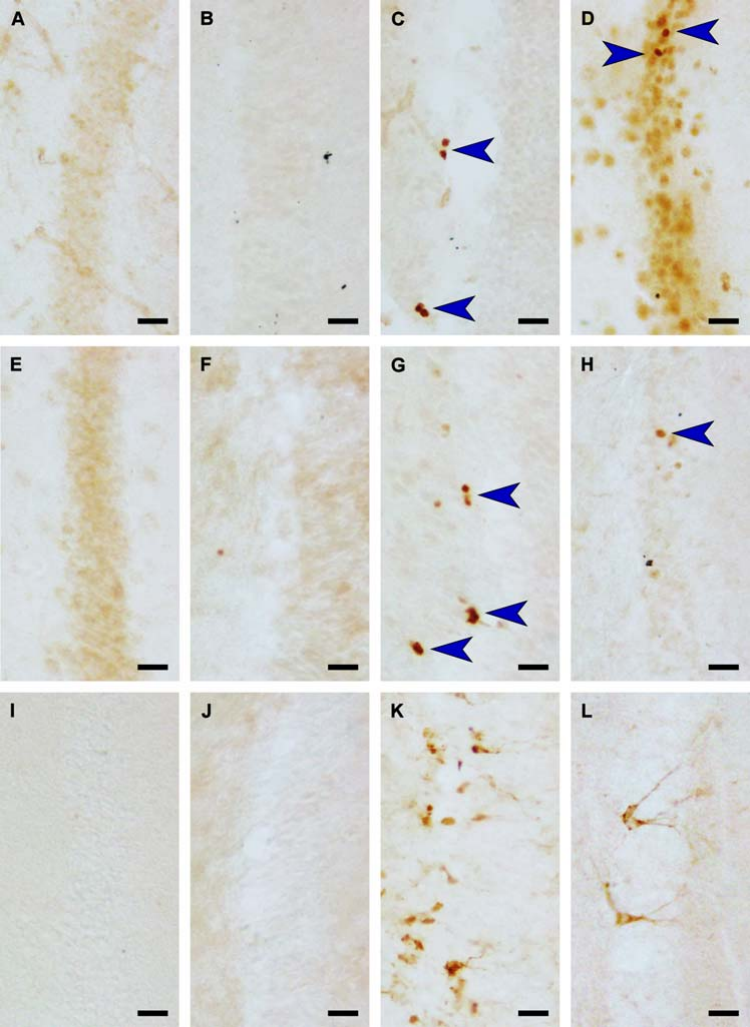
Proliferating and migrating young neurons in the hippocampus of four representative bat species. In the dentate gyrus of nectar and fruit eating *Phyllostomus discolor* (A,E,I) as well as in the insectivorous *Hipposideros cyclops* (B,F,J) we did not detect any proliferating cells with antibodies against Ki-67 (A,B) and MCM2 (E,F), no migrating new neurons can be found with antibody agains DCX (I,J). In contrast, in the sister species *Hipposideros caffer* (C,G,K) and in *Mops condylurus* (D,H,L), proliferating as well as migrating cells can be detected in the subgranular layer of the hippocampus (Ki-67: C,D; MCM2: G,H; DCX: K,L) Molecular layer in all examples on the right side of the granule cell layer, arrows indicate immuno­positive cells. Scale bar is 20µm.

Proliferating cells, detected with Ki-67 and MCM2, in the subgranular layer of the dentate gyrus were absent in *Hipposideros cyclops* (Fig. 2B, F), *Nycteris thebaica* and *macrotis, Neoromicia rendalli, Scotophilus leucogaster* and *Pipistrellus guineensis*. In the Neotropical bats (*Phyllostomus discolor* (Fig. 2A, E), *Glossophaga soricina* and *Carollia perspicillata*), we found none or occasionally one proliferating cell per section within the dentate gyrus proper. We found sparse proliferation activity in *Mops condylurus* (Fig. 2D, H) and *Chaerophon pumila. Hipposideros caffer* (Fig. 2C, G) showed moderate proliferation activity in the subgranular layer. With MCM2 antibody we tested whether bats harbour quiescent precursor cells in the hippocampus. Although MCM2 antibody recognized slightly more positive cells than Ki-67, we did not observe MCM2 positive cells in animals with no Ki-67 staining (Table 1).

**Table 1:** Summary of investigated animals and qualitative immunohistochemical results

Family	Species	N =	Mean BW	Hippocampus		Hippocampus	SVZ/RMS		SVZ/RMS
				Ki-67	MCM2	DCX	KI-67	MCM2	DCX
*Phyllostomidae*	*Glossophaga soricina*	2 (m)	10 g	0	0	0	*	*	*
*Phyllostomidae*	*Carollia perspicillata*	6 (3m/3f)	25 g	0	*	0	**	**	***
*Phyllostomidae*	*Phyllostomus discolor*	2 (f)	45 g	0	0	0	***	***	***
*Hipposideridae*	*Hipposideros caffer*	2 (m)	7 g	**	**	***	***	**	***
*Hipposideridae*	*Hipposideros cyclops*	1 (m)	29 g	0	0	0	**	*	**
*Molossidae*	*Chaerophon pumila*	3 (m)	9 g	*	*	**	**	0	***
*Molossidae*	*Mops condylurus*	6 (m)	30 g	*	*	**	**	**	***
*Nycteridae*	*Nycteris thebaica*	1 (f)	10 g	0	0	0	*	*	0
*Nycteridae*	*Nycteris macrotis*	6 (m)	15 g	0	0	0	***	**	*
*Vespertilionidae*	*Neoromicia rendalli*	1 (m)	10 g	0	–	0	*	**	*
*Vespertilionidae*	*Pipistrellus guineensis*	1 (m)	3.5 g	0	–	0	**	**	**
*Vespertilionidae*	*Scotophilus leucogaster*	1 (m)	17 g	0	0	0	**	**	*

Footnotes: f female; m male; BW body weight; SVZ subventricular zone; RMS rostral migratory stream; *** high immunopositive signal; ** moderate immunopositive signal; * low immunopositive signal; 0 no immunopositive signal; – no data collected;

### Neuronal fate of proliferating cells confirmed with Doublecortin

With Doublecortin (DCX), we detected young migrating neurons in the subgranular layer of the hippocampus only in *M. condylurus* (Fig. 2L), *C. pumila* and *H. caffer* (Fig. 2K). The two molossid species showed low to moderate numbers of new neurons in the caudal (temporal) hippocampus but none or only low levels in the rostral (septal) part. We found the highest levels of DCX positive cells in the hippocampus of *H. caffer* (Fig. 2K). There were no DCX positive cells in the hippocampus of the Neotropical bats (Fig. 2I, Table 1), which indicates that the few proliferating cells detected with Ki-67 and MCM2 may have a glial fate.

### Adult neurogenesis outside the hippocampus

**Figure 3:**
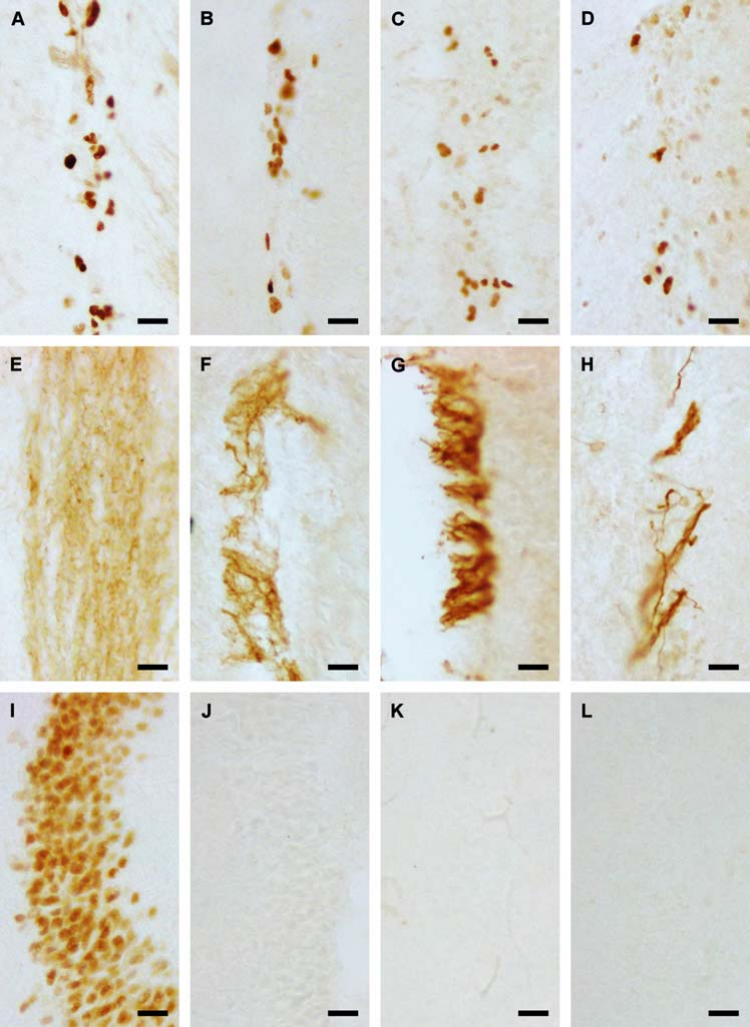
Neurogenesis is not abolished in the rostral migratory stream; NeuroD immunoreactivity has an irregular distribution. Examples of proliferating cells in the rostral migratory stream detected with antibody against Ki-67 (A–D) as well as their neuronal fate visualized with DCX (E–H) are illustrated for *Phyllostomus discolor* (A,E), *Hipposideros cyclops* (B,F), *Hipposideros caffer* (C,G), and *Mops condylurus* (D,H). Thus, animals with and without hippocampal neurogenesis do not differ in their neurogenetic activity in the rostral migratory stream. In *Phyllostomus discolor*, all granule cells in the hippocampus are positive for NeuroD (I), in *Hipposideros cyclops* (J), *Hipposideros caffer* (K) and *Mops condylurus* (L) no reactivity to the antibody against NeuroD could be detected. Scale bar is 20µm.

We detected moderate to ample proliferating cells (Fig. 3 A–D, Ki-67 positive cells) and migrating young neurons (Fig. 3 E–H, DCX positive cells) in the rostral migratory stream in all species. In *N. macrotis, N. rendalli, S. leucogaster* and *G. soricina* few DCX positive cells were present in the rostral migratory stream and the olfactory bulb. In *N. thebaica*, the antibody against DCX could not detect any antigen (Table 1).

### Differentiation of granule cells

All hippocampal granule cells were homogeneously positive for NeuroD in *P. discolor* (Fig. 3I) and *S. leucogaster*, slightly less intensely so in *G. soricina* and *C. perspicillata*. No NeuroD was detected in granule cells of *H. caffer* (Fig. 3K), *H. cyclops* (Fig. 3J), *C. pumila*, *M. condylurus* (Fig. 3L), *N. thebaica* and *N. macrotis*. For *N. rendalli* and *P. guineensis* no NeuroD immunoreactivity data was obtained.

## Discussion

In nine out of twelve African (Paleotropical) and Central/South American (Neotropical) bat species we found no indication for young neurons in the dentate gyrus of the hippocampus. Due to small sample size in some of the species, our data have preliminary character. However, positive staining controls in the brains of all bats indicate that our negative findings in the hippocampus are not due to inappropriate methodology. The large proportion of bat species without apparent adult neurogenesis in the hippocampus is surprising. It indicates that in the second-largest mammalian order after rodents, functionality of the adult hippocampus in terms of large-scale spatial behavior does not necessarily require neurogenesis. Bats appear to share low rates of adult neurogenesis with some large-sized primates, including humans. While our data provide a counter-example to some widely held views derived from observation in rodents, they may be helpful in developing novel views in understanding the physiological role of adult neurogenesis.

### Low rates of adult neurogenesis in bats do not reflect problems in immunohistochemical sensitivity

**Figure 4:**
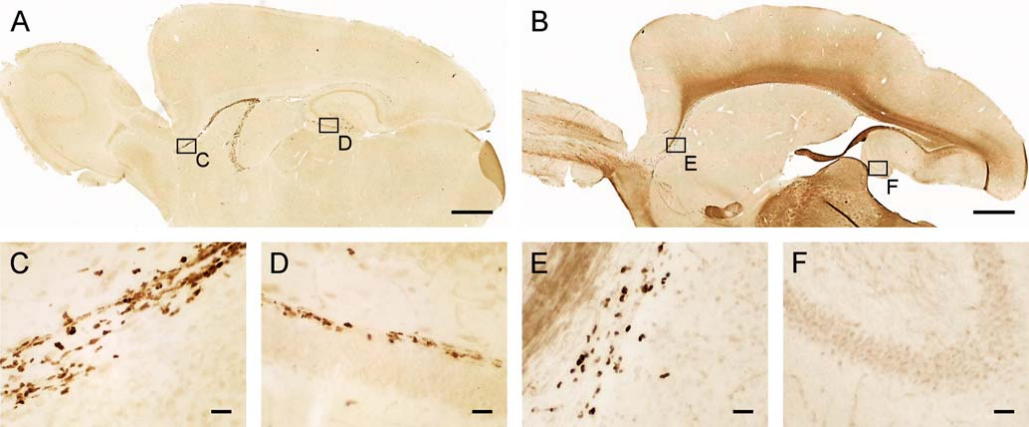
Mice and bats show similar proliferation activity in the RMS, but not in the hippocampus. Immunohistochemistry against Ki-67 in wild trapped adult wood mouse (*Apodemus flavicollis*: A,C,D) shows intense proliferation activity in the RMS (A, insert C) as well as in the subgranular layer of the dentate gyrus (A, insert D). The same protocol applied to a neotropical bat (*Phyllostomus discolor*: B,E,F) visualizes a continuous band of proliferating cells along the RMS (B, insert E), but proliferating cells are completely missing in the granule cell layer (B, insert F). Scale bar in A,B is 1mm, in C-F 25µm.

An obvious concern in comparative studies using immunohistochemical mapping of proteins is whether the technique employed misses species-specific epitopes, thus providing false negative data. However, this is almost certainly not the case here. (i) Adult neurogenesis has been assessed by different cell markers indicating proliferation (Ki-67), juvenile stages of neurons (DCX), and slowly dividing precursor cells (MCM2). Both Ki-67 and MCM2 are evolutionarily highly conserved proteins that have thus far been found in all vertebrate species investigated [14], [17]. (ii) We have employed a standardized procedure that has been used for comparative studies of various small rodent species, in which differences in rates of adult neurogenesis could be detected reliably [12]. (iii) Most importantly, the same immunohistochemical procedure visualized numerous immunopositive cells in the subventricular zone and the rostral migratory stream in bats (Fig. 4 B, close up E) and mice (Fig. 4 A, and close up C), whereas proliferating, Ki-67 positive cells in the same sections can be found in the mouse subgranular layer (Fig. 4, D) but not in those of the bat (Fig. 4, F). The staining pattern as shown in Figure 4 indicates that missing or scarce proliferation activity in the granule cell layer is restricted to the hippocampal formation in bats. In rodents, a widely used technique for labeling dividing cells is the immunohistochemical visualization of the intraperitoneally injected thymidine analogue bromodeoxyuridine (BrdU) that is incorporated in to the DNA during mitosis. BrdU is cleared from rat brains within a short phase of 2 hours [18]. Endogenous Ki-67 is expressed during active cell cycle, which has been estimated to last for 12–14 hours in mice [19]. Thus, the use of BrdU would require repeated injections and housing of the animals for at least 14 hours in order to obtain the same sensitivity as the labeling technique employed by us, which can be applied to brains of animals sacrificed immediately after capture.

### Low levels of adult hippocampal neurogenesis across bats do not permit correlative analysis with ecological parameters yet

Previous studies have indicated that the relative size of the hippocampus in bats might be correlated to habitat size, diet or foraging strategy. Clearly, missing adult hippocampal neurogenesis or low levels thereof show that between-species variations of this trait are not crucial for a variety of functions and behaviors thought to depend on the hippocampus of bats, unless one assumes that minor differences in the proliferation rate of granule cells might be functionally important. For example, *Hipposideros caffer* with the relatively highest amount of adult neurogenesis in our sample uses small to medium sized territories, while species with sparse or no adult neurogenesis include both, species with very small home ranges (*Hipposideros cyclops*) [20]) and species with comparatively large home ranges (e.g. *Phyllostomus discolor, Chaerophon pumila*). Our sample contains no species known for long distance migration, and ecological data on some of the species in our sample are incomplete or missing. However, the observation that nectar-eating bats with highly developed spatial reference memory (knowing where to go) and spatial working memory (knowing which host plant has been visited and when) [21], [22] demonstrates that such cognitive abilities do not depend on adult cell proliferation in the hippocampus. Assuming that the hippocampus is indeed crucially involved in spatial navigation and memory of bats, this observation would imply that adult hippocampal neurogenesis is not of general necessity for superior performance in spatial abilities.

### Seasonal variations, longevity and neurogenesis

A possible source for missing neurogenesis might be seasonal variations, as observed, for example, in the song control nuclei of songbirds [23]. Investigations of wild-living squirrels captured over the year reveal no difference in proliferation activity, despite considerable behavioral adaptation due to seasonally changing needs for spatial learning and memory [24]. Given that our data are from tropical bats, it would seem rather unlikely to expect seasonal variations in adult hippocampal neurogenesis yet sparing the SVZ and RMS. Cell proliferation in the hippocampus, always highest in subadult individuals, is known to decline with age in wild as well as laboratory rodents [12], [25], and also in humans [26]. It thus appears to be the most general constraint on adult hippocampal neurogenesis. Bats live up to 3.5 times longer than other mammals of comparable size [27]. The Neotropical bats in our sample are reported to have wild live spans between 9 (*P. discolor*), 10 (*G. soricina*) and 12.4 years (*C. perspicillata*), which is much longer than in same sized rodents, and close relatives of the three species showing neurogenesis have similar life spans as the Neotropical species [27]. Due to the difficulty of accurate age determination in adult bats, our samples probably contain young adults as well as older animals. However, in the species where we investigated several individuals we did not observe any marked individual differences in terms of neurogenesis activity. This implies that we have not been investigating selectively old animals, and that the paucity of proliferating cells occurs already in adult and not only in old animals.

### Species comparisons of adult hippocampal neurogenesis: what can we learn from negative findings?

Based on our findings in wild-living rodents, and current ideas about the functional role of adult hippocampal neurogenesis, we undertook this study expecting to find high rates of neurogenesis in bats, hopefully co-varying with ecological parameters so manifold in Chiroptera. While the missing or low proliferation rates in many bat species clearly impede the search for ecological covariates of adult hippocampal neurogenesis (yet not for other co­varying brain traits), they also require a re-consideration of some widely held beliefs in the field. These beliefs tend to neglect or downplay comparative issues, and are often based on a “more-is-better” view of adult hippocampal neurogenesis. Yet, out of negative findings, interesting functional and clinical hypotheses and approaches may emerge.

#### Behavioral flexibility and adult neurogenesis?

From a comparative point of view, we hypothesize that high and unrestricted rates of proliferation in the adult hippocampus might be a feature of short-living mammals, where neurogenesis may be affordable and sufficient to supply the plasticity functions attributed to newly generated cells throughout a fairly short life span. During their short life, most rodents are strongly predated upon, requiring permanent adjustment of spatial relations and behavioral reactions, in short, high levels of behavioral flexibility throughout life, particularly as related to avoidance and escape behavior. Assuming longevity and lower ecological predation pressure for most bats and certainly humans, these species might afford to sacrifice behavioral flexibility regarding spatial abilities, relying instead on an established set of memory relations between objects and places. In humans, this could correspond to the well-known (and debated) transition from juvenile “fluid” intelligence to the “crystallized” intelligence of middle-aged and elderly persons [28], [29]. Whether physiological adult hippocampal neurogenesis in humans is related to different forms of intelligence is likely to remain an open question. On the other hand, loss of spatial behavioral flexibility in humans as indicated by path routines is an often observed event in life history. Likewise, many bat species use stereotypical flight paths when commuting between roosts and foraging areas permitting researchers to catch them there with nets. Even the nectar-eating bats with their excellent spatial memory need hundreds to thousands of trials to attain object-shape discrimination when the objects change location [30], while mice can learn conceptually related tasks in 50–100 trials [31]. Bats and primates, including humans [32], [33] may share the trait for low rates of adult neurogenesis when compared to rodents.

#### Loss of function or down-regulation?

Recent investigations in healthy adult humans using endogenous markers showed missing or low ongoing proliferation in the hippocampus, whereas in the young individuals in these studies proliferation activity could always be detected [26], [34]–[36]. At first glance, this appears to be an age-dependent loss-of-function phenomenon in species with long life span. However, it is not unlikely that sparse or missing adult hippocampal neurogenesis is actually caused by down-regulation of mitogenic activity in the subgranular zone, as proliferation is regularly observed in the SVZ , RMS and olfactory bulb of bats (Fig. 3, A–H; Fig. 4, B,E) and, possibly, in humans [37]–[39]. Proliferation of new cells in the mammalian brain can also be triggered by insults [40], suggesting likewise the presence of hippocampus-specific down-regulating factors. For clinically oriented neuroscience, such relations would imply that bat hippocampi of any other than juvenile age may correspond functionally to those of middle-aged or elderly humans. This bears the promise of using, selectively and focused, some bat species as animal models corresponding better to the human condition, particularly when searching for regulatory factors. Clearly, more comparative studies are needed, not only in bats, to identify species that model the characteristic of human adult hippocampal neurogenesis.

### Conclusions and outlook

- 1 Low rate (25%) or missing (75%) adult hippocampal neurogenesis in twelve bat species with different home ranges, foraging habits and relative hippocampal sizes indicate that such proliferation is not necessary for their home-range spatial behavior, pending investigations in migratory bats.
- 2 This result was not a methodological artifact since persisting proliferation in the subventricular zone and the presence of a rostral migratory stream of young neurons was clearly demonstrated.
- 3 The wide ecological radiation and a corresponding range of behavioral and neural adaptations within the order Chiroptera offers excellent possibilities to test empirically evolutionary adaptations predicted by findings from other species.
- 4 The most interesting correlate of low or absent adult hippocampal neurogenesis is the remarkable longevity of bats; low rates of adult neurogenesis combined with long life spans are also found in monkeys and humans.
- 5 Given this similarity, bats might prove to be a useful animal model for analyzing the functionality of adult neurogenesis and associated cellular processes in humans, be this for natural or pathological conditions. An interesting possibility might be the search for local down-regulating factors
- 6 This study underlines the necessity to investigate the phenomenon of adult hippocampal cell proliferation across many species if a more complete understanding of evolutionary mechanism in neuronal plasticity in mammals is to be reached. This is not to deny the usefulness of the widespread mouse or rat models, but species-specific peculiarities must be assessed across a broader range of species.

## Material and Methods

### Animals

Neotropical bats were provided from breeding colonies at the University of Munich, Germany. African animals were trapped in Forêt Classée de la Lama in the Zou province, Republic of Benin, West Africa using standard bat trapping techniques and perfused rapidly after trapping. All animals in the sample were adult but their exact ages are unknown. In the field, animals were identified as adults by confirming complete closure of the epiphyseal growth plates in the metacarpal-phalangeal joint of the finger bones [41]. All research in Benin, export of brains and specimens to Switzerland was covered by permits of the Faculté des Sciences Agronomiques, Université d'Abomey-Calavi (FSA-UAC) and was in concordance with the laws of the Republic of Benin. None of the captured species are threatened or protected (www.redlist.org). For complete list of animals see Table 1. All experimental procedures were conducted in accordance with the Swiss animal welfare guidelines for the care and use of laboratory animals and approved by the cantonal veterinarian office of Zürich, Switzerland.

### Neurohistology

Animal where anesthetized intraperitoneally with Nembutal and perfused transcardically with phosphate buffered saline (PBS), followed by 0.6% sodium sulphide solution and 4% paraformaldehyde. Brains were removed and postfixed over night. After saturation with 30% sucrose solution, brains were frozen. Coronal cryostat sections (40 µm) in African and sagittal sections (40 µm) in American bats of right hemispheres were collected in series of 4, 6 or 12 according to brain size and used for free floating immunohistochemistry. Sections were rinsed in Tris buffered saline (TBS) containing 0.05% Triton. For epitope retrieval, tissue was incubated in citric buffer, pH 6.4 for 40 minutes at 95°C, rinsed again and incubated for 1 hour in TBS containing 0.25% Triton, 2% normal serum of the animal the secondary antibody was raised in, and 1% bovine serum albumin (BSA). Primary antibodies MCM2 (polyclonal goat antibody, Santa Cruz Biotechnology 1∶500), Ki-67 (NCL-Ki-67p, polyclonal rabbit antibody, Dianova 1∶1000) and NeuroD (polyclonal goat IgG, Santa Cruz Biotechnology 1∶500) were diluted in the same diluent, and sections were incubated at 4°C over night. Incubation with secondary antibodies (rabbit anti goat, goat anti rabbit Vectastain Elite ABC kit) was followed by avidin-biotin complex according to manufacturer's instructions and stained with DAB as chromogen. For Doublecortin (DCX, polyclonal goat antibody, Santa Cruz Biotechnology, 1∶1000) sections were not heat treated. Endogenous peroxidase activity was blocked by incubation of the sections in 0.6% hydrogen peroxidase for 30 minutes. All other steps followed the protocols described above. Sections were mounted, embedded and investigated on an Olympus BX 40 microscope using 20× and 40× objectives. For each marker and animal, between 2 and 12 sections containing the hippocampal structure were analyzed qualitatively. Immunohistochemical visualization of cell proliferation by means of Ki-67 has been validated against the bromodeoxyuridine labeling technique elsewhere [42], showing high correlations between the two methods.

## References

[1] Fleming TH, Eby P, Kunz TH, Fenton MB (2003). Ecology of bat migration.. Bat ecology.

[2] Wilson MA, McNaughton BL (1993). Dynamics of the hippocampal ensemble code for space.. Science.

[3] Ulanovsky N, Moss CF (2007). Hippocampal cellular and network activity in freely moving echolocating bats.. Nature Neurosci.

[4] Baron G, Stephan H, Frahm HD (1996). Comparative Neurobiology in Chiroptera: Macromorphology, brain structures, tables and atlases.

[5] Safi K, Dechmann DK (2005). Adaptation of brain regions to habitat complexity: a comparative analysis in bats (Chiroptera).. Proc R Soc B.

[6] van Praag H, Schinder AF, Christie BR, Toni N, Palmer TD (2002). Functional neurogenesis in the adult hippocampus.. Nature.

[7] van Praag H, Kempermann G, Gage FH (1999). Running increases cell proliferation and neurogenesis in the adult mouse dentate gyrus.. Nature Neurosci.

[8] McEwen BS (1999). Stress and hippocampal plasticity.. Annu Rev Neurosci.

[9] Ming G, Song H (2005). Adult neurogenesis in the mammalian central nervous system.. Annu Rev Neurosci.

[10] Leuner B, Gould E, Shors TJ (2006). Is there a link between adult neurogenesis and learning?. Hippocampus.

[11] Amrein I, Slomianka L, Lipp HP (2004). Granule cell number, cell death and cell proliferation in the dentate gyrus of wild-living rodents.. Eur J Neurosci.

[12] Amrein I, Slomianka L, Poletaeva, Bologova NV, Lipp HP (2004). Marked species and age-dependent differences in cell proliferation and neurogenesis in the hippocampus of wild-living rodents.. Hippocampus.

[13] Starborg M, Gell K, Brundell E, Höög C (1996). The murine Ki-67 cell proliferation antigen accumulates in the nucleolar and heterochromatic regions of interphase cells and at the periphery of the mitotic chromosomes in a process essential for cell cycle progression.. J Cell Sci.

[14] Tye BK (1999). MCM proteins in DNA replication.. Annu Rev Biochem.

[15] Matsuo N, Kawamoto S, Matsubara K, Okubo K (1998). Cloning and developmental expression of the murine homolog of doublecortin.. Biochem Biophys Res Commun.

[16] Lee JE (1997). Basic helix-loop-helix genes in neural development.. Curr Opin Neurobiol.

[17] Scholzen T, Gerdes J (2000). The Ki-67 protein: from the known and the unknown.. J Cell Physiol.

[18] Cameron HA, McKay RD (2001). Adult neurogenesis produces a large pool of new granule cells in the dentate gyrus.. J Comp Neurol.

[19] Hayes NL, Nowakowski RS (2002). Dynamics of cell proliferation in the adult dentate gyrus of two inbred strains of mice.. Brain Res Dev Brain Res.

[20] Decher J, Fahr J (2005). *Hipposideros cyclops*.. Mamm species.

[21] Thiele J, Winter Y (2005). Hierarchical strategy for relocating food targets in flower bats: spatial memory versus cue-directed search.. Anim Behav.

[22] Winter Y, Stich KP (2005). Foraging in a complex naturalistic environment: capacity of spatial working memory in flower bats.. J Exp Biol.

[23] Alvarez-Buylla A, Kirn JR (1997). Birth, migration, incorporation, and death of vocal control neurons in adult songbirds.. J Neurobiol.

[24] Lavenex P, Steele MA, Jacobs LF (2000). The seasonal pattern of cell proliferation and neuron number in the dentate gyrus of wild adult eastern grey squirrels.. Eur J Neurosci.

[25] Kuhn HG, Dickinson-Anson H, Gage FH (1996). Neurogenesis in the dentate gyrus of the adult rat: age-related decrease of neuronal progenitor proliferation.. J Neurosci.

[26] Fahrner A, Kann G, Flubacher A, Heinrich C, Freiman TM (2007). Granule cell dispersion is not accompanied by enhanced neurogenesis in temporal lobe epilepsy patients.. Exp Neurol.

[27] Wilkinson GS, South JM (2002). Life history, ecology and longevity in bats.. Aging Cell.

[28] Belsky JK (1990). The psychology of aging: theory, research and interventions.

[29] Horn JIE, Goulet R, Baltes PB (1970). Organization of data on life-span development of human abilities.. Life-span developmental psychology: Research and theory.

[30] Stich KP, Winter Y (2006). Lack of generalization of object discrimination between spatial contexts by a bat.. J Exp Biol.

[31] Lipp H-P, Van der Loos H (1991). A computer-controlled Y-maze for testing vibrisso­tactile discrimination learning in mice.. Behav Brain Res.

[32] Eriksson PS, Perfilieva E, Bjork-Eriksson T, Alborn AM, Nordborg C (1998). Neurogenesis in the adult human hippocampus.. Nature Med.

[33] Gould E, Reeves AJ, Fallah M, Tanapat P, Gross CG (1999). Hippocampal neurogenesis in adult Old World primates.. Proc Natl Acad Sci USA.

[34] Boekhoorn K, Joels M, Lucassen PJ (2006). Increased proliferation reflects glial and vascular-associated changes, but not neurogenesis in the presenile Alzheimer hippocampus.. Neurobiol Dis.

[35] Del Bigio MR (1999). Proliferative status of cells in adult human dentate gyrus.. Microsc Res Tech.

[36] Seress L, Abraham H, Tornoczky T, Kosztolanyi G (2001). Cell formation in the human hippocampal formation from mid-gestation to the late postnatal period.. Neuroscience.

[37] Bedard A, Parent A (2004). Evidence of newly generated neurons in the human olfactory bulb.. Brain Res Dev Brain Res.

[38] Curtis MA, Penney EB, Pearson J, Dragunow M, Connor B (2005). The distribution of progenitor cells in the subependymal layer of the lateral ventricle in the normal and Huntington's disease human brain.. Neuroscience.

[39] Sanai N, Tramontin AD, Quinones-Hinojosa A, Barbaro NM, Gupta N (2004). Unique astrocyte ribbon in adult human brain contains neural stem cells but lacks chain migration.. Nature.

[40] Lichtenwalner RJ, Parent JM (2006). Adult neurogenesis and the ischemic forebrain.. J Cereb Blood Flow Metab.

[41] Anthony ELP, Kunz TH (1988). Age determination in bats.. Ecological and behavioral methods for the study of bats.

[42] Kee N, Sivalingam S, Boonstra R, Wojtowicz JM (2002). The utility of Ki-67 and BrdU as proliferative markers of adult neurogenesis.. J Neurosci Meth.

[43] Teeling EC, Springer MS, Madsen O, Bates P, O'Brien SJ (2005). A molecular phylogeny for bats illuminates biogeography and the fossil record.. Science.

